# Effectiveness of Computer-Assisted Therapy for Substance Dependence Using Breaking Free Online: Subgroup Analyses of a Heterogeneous Sample of Service Users

**DOI:** 10.2196/mental.4355

**Published:** 2015-04-23

**Authors:** Sarah Elison, Glyn Davies, Jonathan Ward

**Affiliations:** ^1^ Breaking Free Online Manchester United Kingdom

**Keywords:** substance misuse, computer-assisted therapy, treatment, psychosocial interventions

## Abstract

**Background:**

Substance misuse services within the United Kingdom have traditionally been oriented to opiate and crack users, and attended predominantly by male service users. Groups who do not fit this demographic, such as women or those whose primary drug of choice is neither heroin nor crack, have tended to be underrepresented in services. In addition, there can be stigma associated with traditional opiate and crack-centric services. Therefore, the computerized treatment and recovery program, Breaking Free Online (BFO), was developed to enable service users to access confidential support for dependence on a wide range of substances. BFO is delivered as computer-assisted therapy (CAT), or, where appropriate, used as self-help.

**Objective:**

The aim of this study was to report psychometric outcomes data from 393 service users accessing online support for substance misuse via BFO.

**Methods:**

Following initial referral to substance misuse services, all participants were supported in setting up a BFO login by a practitioner or peer mentor, and, where required, assisted as they completed an online baseline assessment battery contained within the BFO program. Following a period of engagement with BFO, all participants completed the same battery of assessments, and changes in the scores on these assessments were examined.

**Results:**

Significant improvements were found across the 393 service users in several areas of psychosocial functioning, including quality of life, severity of alcohol and drug dependence, depression, and anxiety (*P*=<.001 across all aspects of functioning)*.* Additionally, significant improvements were found within specific subgroups of participants, including females (*P*=.001-<.001), males (*P*=.004-<.001), service users reporting alcohol dependence (*P*=.002-<.001), opiate and crack dependence (*P*=.014-<.001), and those seeking support for other substances that may be less well represented in the substance misuse sector (*P*=.001-<.001).

**Conclusions:**

Data from this study indicates that BFO is an effective clinical treatment for a wide range of individuals requiring support for substance misuse. Further work is currently underway to examine more closely the clinical effectiveness of the program.

## Introduction

In recent years, the substance misuse sector has been subject to changes that may have long-term implications for societal attitudes to substance misuse, and the ways in which individuals facing such difficulties may best be supported [1]. For example, there seems to be increasing recognition that the range of substances of misuse and dependence may be wider than alcohol, opiates, and crack. Regulatory bodies such as the National Institute for Health and Care Excellence (NICE) and the National Treatment Agency (NTA) have published guidelines around the treatment of cannabis and stimulants [2], and prescription and over the counter medication [3] dependencies. However, substance misuse services have not traditionally been designed for non-opiate or crack-using individuals [4]. Such services have been designed primarily to provide support to opiate and crack-using individuals, who represent the more traditionally identifiable substance dependent groups within society. Although services may have some provisions for non-opiate or crack-using individuals, they continue to be perceived as being oriented towards opiate and crack dependencies [5].

There is growing interest in the psychosocial issues that drive substance dependence, with dependence now being seen by some as a symptom of underlying mental health issues [6], relationships [7], lifestyle [8], and other social and economic difficulties [9]. Consequently, attitudes appear to be changing regarding the possibility of someone recovering from substance dependence, as previous conceptions of dependence as a chronic relapsing condition are being challenged [10-13].

In addition to non-opiate and crack-using individuals, women are another demographic that though may be presenting at services more frequently, albeit still underrepresented. Women may find it particularly difficult to access services, often due to the risks associated with the involvement of social services when dependent children are involved [14]. Despite this underrepresentation, recent estimates demonstrate that much higher proportions of women may be dependent on substances than come into contact with services [4]. As well, the number of women in the United Kingdom consuming alcohol to hazardous, harmful or dependent levels, and the rate of alcohol-related deaths among women is also increasing [15,16]. One major barrier to such individuals accessing substance misuse services may be the stigma associated with seeking support for substance dependence, especially when such services may still viewed by some as being opiate and crack-centric.

Thus, there appears to be changes in both substance use patterns and the types of groups requiring support from substance misuse services. Therefore, there is a need for evidence-based, clinically effective intervention approaches that are appropriate for addressing a wide variety of forms of substance misuse and dependence, and the mental health and wider psychosocial issues that underpin them.

Although there have been interventions developed to address substance misuse and co-occurring mental health issues [17], few address mild to moderate mental health difficulties, and instead target more severe mental health issues. Despite being reported in the literature as effective, there are challenges in getting these interventions commissioned within the substance misuse sector and making them available to the general public [18]. However, such intervention approaches may be beneficial as they provide support in such a way that helps to overcome barriers to access due to stigma, especially if individuals requiring support from such services may not necessarily identify themselves as having a mental illness (ie, mild or temporary mental health issues).

One potential solution to these requirements, in terms of reducing barriers to accessing services for individuals with mild to moderate mental health difficulties, is Breaking Free Online (BFO), an online psychosocial intervention that has been developed and commissioned in over 60 local authorities in UK-based substance misuse services [19-22].

BFO can be delivered as computer-assisted therapy (CAT), or as self-help, and is designed to support people in their recovery from substance misuse. BFO delivers evidence-based psychosocial intervention strategies that are compliant with NICE guidance around interventions for substance misuse [23], offers a range of different multimedia formats, and specifically targets 36 different substances (ie, substitute medications, legal highs, and prescribed medications of abuse).

**Figure 1:**
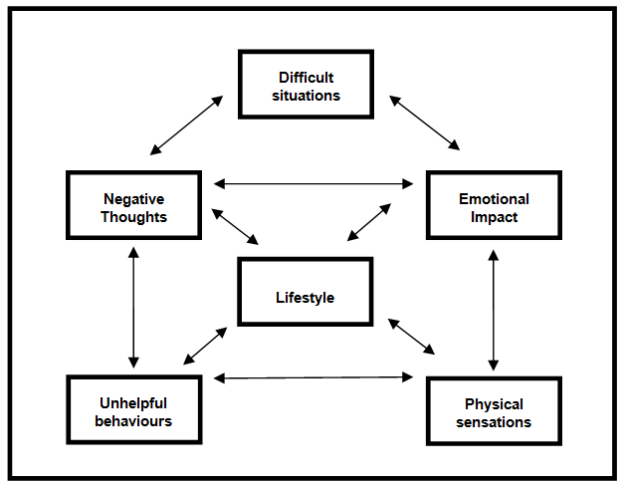
Domains of functioning contained within the LBM.

The BFO program provides access to 22 interactive, evidence-based intervention strategies taken from cognitive-behavioral therapy (CBT) [24] and mindfulness approaches [25]. Audio and visual technology is used to deliver intervention content that has traditionally been delivered with service users via face to face interactions with a practitioner or paper-based documents. The content of the program was developed through consultation with substance misuse and mental health professionals and service users, and a review of the literature around evidence-based approaches for substance misuse. All intervention content is structured around a six domain model that conceptualizes various aspects of biopsychosocial functioning associated with substance misuse and any comorbid mental health difficulties. The model, the Lifestyle Balance Model (LBM) [22], was developed by the authors of this study and is based on the commonly used five-factor model used in mental health case formulation [26,27]. The LBM offers a guided node-link map structure for understanding an individual’s substance use and associated difficulties, irrespective of the type of substance difficulty [28,29]. The domains of functioning contained within the LBM are depicted in Figure 1.

An assessment within the program, the Recovery Progression Measure (RPM) [submitted], was developed by the authors of this study to measure functioning in the six LBM domains. The RPM is an online-based series of assessments that can be completed by service users with support from a professional or alone. The assessments are completed at intervals to provide follow-up data. Data from the program are stored on a secure, offsite server, and security protocol conforms to Caldicott guidance [30], and other relevant data protection and legal requirements. All stored data are also completely confidential and contain no identifiable service user information.

Initial evaluations indicated that the BFO program significantly reduced substance misuse, and improved mental health and quality of life [20,21]. As well, there is evidence to support the modality of the delivery and therapeutic components within the program [25,31,32]. Accessing online interventions, like BFO, may help to overcome barriers such as the shame and stigma sometimes associated with accessing more visible, traditional opiate and crack- centric drug and alcohol services [33]. It can also ensure that access is both confidential and anonymous.

This study was an outcomes evaluation of the effectiveness of BFO on a heterogeneous sample of 393 service users accessing support for substance dependence via substance misuse services in the United Kingdom. In addition to assessing the effectiveness of BFO for the group as a whole, subgroup analyses were reported around the effectiveness of previously less represented groups in services, such as women, and service users seeking support for dependence on substances other than opiates and crack, including alcohol.

## Methods

### Design

This is a quantitative, repeated-measures, psychometric outcomes study that reports data from 393 service users presenting to drug and alcohol services accessing support for substance dependence via BFO.

### Participants

Participants were from a group of 785 service users accessing an updated version of BFO released in January 2013, who had all completed a baseline assessment, and logged onto the program to access at least one intervention strategy during the period from January-December, 2013. Of this group of 785 service users, 393 completed a follow-up post-intervention assessment providing data to the study, resulting in a response rate of 50.0% (393/785). All participants had either self-referred or been referred into a specialist substance misuse service by a health or social care professional (eg, general practitioner or social worker), or via the criminal justice system (eg, police or probation). All participants were provided free access to BFO from the referring service. Ethical approval was previously granted for using the Breaking Free Online research database containing service user assessment data (application ‘Breaking Free Online Research Database'; Research Ethics Committee Reference 12/LO/0076). Participants were not given any incentive to either use the BFO program or provide data for the study.

A total of 224 (57.0%, 224/393) participants were male, and the mean age was 42.4 years (range 15-73 years, SD 11.3). The group was predominantly White-British/White-Irish (95.9%, 377/393), with the rest being Asian/Asian-British (1.3%, 5/393), Black/Black-British (0.3%, 1/393), mixed-race (2.5%, 10/393), or other unspecified ethnicities (0.3%, 1/393). With respect to the severity of the drug dependence at baseline, of those reporting an illicit substance as their principal substance of dependence, a total of 169 participants (92.9%, 169/182) reached the cut-off for clinically significant drug dependence by scoring ≤3 on the Severity of Dependence Scale (SDS). For those 299 reporting alcohol as being one of their substances of dependence (with 89 of these also reporting being dependent on an illicit drug as well as alcohol), 259 participants (86.6%, 259/299) reached cut-off for clinically significant alcohol dependence. A total of 275 participants (70.0%, 275/393) reached clinical cut-off for clinically significant depression by scoring ≤4 on the Patient Health Questionnaire (PHQ), and of those who reported some difficulties with anxiety, 89 (69.0%, 89/129) reached cut-off for clinically significant anxiety by scoring ≤4 on the General Anxiety Disorder (GAD) scale.

A wide range of substances were reported by participating service users as being the main problem substance, from prescribed medications, to opiate substitute medications, and synthetic psychoactive substances, however, alcohol was the most common (53.4%, 210/393), followed by heroin (13.2%, 52/393), and cannabis (11.7%, 46/393). In total, approximately a quarter (24.2%, 95/393) cited non-opiate and crack substances (excluding alcohol) as being their main substance of dependence, and just over one fifth (22.4%, 88/393) cited their main substance of dependence being an opiate (including opiate substitutes) or crack. Full details of the substances used by the sample are provided in Table 1.

**Table 1:** The main substances of dependence (N=19).

Substance		n (%)
**Alcohol**		210 (53.4)
**Non-opiate (and opiate substitute) or crack**		
	Amphetamines	11 (11.0)
	Cannabis	46 (11.7)
	Cocaine	16 (4.1)
	Diazepam	7 (7.0)
	Dihydrocodeine	2 (0.5)
	Etizolam	1 (0.3)
	GBL	1 (0.3)
	Ketamine	2 (0.5)
	Khat	2 (0.5)
	Mephedrone	3 (0.8)
	Temazepam	1 (0.3)
	Tramadol	2 (0.5)
	Zopiclon	1 (0.3)
	Total	95 (24.2)
**Opiate (and opiate substitute) or crack**		
	Buprenorphine	5 (1.3)
	Crack	12 (3.1)
	Heroin	52 (1.2)
	Methadone	18 (4.6)
	Suboxone	1 (0.3)
	Total	88 (22.4)

### Procedure

Following initial referral to services, all participants were supported in setting up a BFO login by a practitioner or peer mentor, and, where required, assisted as they completed an online baseline assessment battery contained within the BFO program. The assessments are shown in Textbox 1.

**Baseline assessments of the BFO program.**

Assessments

- Recovery Progression Measure (RPM) [submitted]
  - A 36-item measure comprising 6 Likert scale items, each with 11 points (ie, 0-10) that determines the impact of difficult situations, negative thoughts, emotions, unhelpful behaviors, physical sensations, and lifestyle on substance use.
  - Contains 30 dichotomous ‘yes/no’ response items that measure the presence or absence of specific psychosocial issues within each of the 6 Likert scale items.
- World Health Organization Quality of Life Measure (WHOQOL-BREF) [34]
  - A total of 5 items (1, 2, 17, 18, and 20) from the WHOQOL-BREF were selected for measuring general quality of life.
- Patient Health Questionnaire (PHQ-9) [35]
  - A 9-item scale that measures the levels of depression, and also contains validated clinical norms.
- General Anxiety Disorder Scale (GAD-7) [36]
  - A 7-item scale that measures the levels of anxiety, and also contains validated clinical norms.
- Severity of Dependence Scale (SDS) [37]
  - A 5-item scale that measures the severity of alcohol dependence.

The RPM was specifically developed by the authors of this study as a tool to measure the degree of ‘recovery progression’ an individual achieves in each domain. The internal reliability of the RPM measure was excellent (alpha>.70), with item-total correlations revealing moderate to excellent reliability of individual items. As well, the convergent validity was excellent, with the RPM measure correlating significantly with scores on standardized psychometric measures of related constructs, such as the SDS [37], PHQ [35], and GAD [36]. Exploratory factor analyses (EFA) revealed the RPM contained an underlying factor structure consisting of eight components.

Upon completion of the baseline assessment, individuals were provided with full access to BFO. Most individuals accessed BFO both within services with support from a practitioner or peer mentor, and at home or in community settings with internet access, such as local libraries. Time periods of engagement with the program varied, reflecting the program’s ability to be tailored to the needs of the individual; some participants engaged for longer periods than others, depending on their perception of need. The amount of engagement with BFO depended on the individual’s perception of how much they felt they needed to use the program in order to address the specific type and severity of difficulties they were experiencing. At the end of each individual’s period of engagement with the program, the same battery of assessments was completed online.

### Analysis

As data were not normally distributed, non-parametric Wilcoxon signed-ranks tests were conducted to examine the changes in psychometric scores from baseline to post-intervention follow up. Effect sizes were also calculated for these, and multiple linear regressions were conducted to examine the association between the time in weeks and changes in psychometric scores.

## Results

Engagement with the BFO program varied by the period of weeks, the total amount of time spent online, and the number of intervention strategies accessed. The mean engagement period was 4.6 weeks (range from 1-12 weeks, SD 3.4), the mean time spent online was 4.7 hours (range 18 minutes-109 hours, SD 7.8), and the mean number of strategies accessed was 6.8 (range 1-12, SD 3.7).

As Shapiro-Wilk tests revealed data to be non-normally distributed (*P*<.05), non-parametric Wilcoxon signed-ranks tests were run to examine possible changes in psychometric scores from baseline to follow-up. Analyses revealed that a number of statistically significant changes were identified both in terms of psychometric scores, and that these were evident in each of the subgroups included in the study. In addition to exploring changes in scores from baseline to post-intervention follow-up, effect sizes were calculated in order to examine the strength of any identified changes in scores. Linear regressions were run to ascertain whether changes in scores were a function of time elapsed in weeks between baseline and follow-up assessments. Outcomes for each of the following groups are reported in Table 2.

**Table 2:** Psychometric outcomes from baseline to post-treatment follow-up (N=393).

Category		Changes in psychometric scores					Linear regression^a^		
		Baseline, mean (SD)	Follow-up, mean (SD)	*z* score	*P* value	Effect size, *r*	*R*	*R* ^*2*^	*P* value
**All data, n=393**									
	Quality of life	8.9 (4.5)	11.0 (4.5)	8.417	<.001	0.43	.065	.004	.198
	RPM	38.2 (14.2)	32.7 (15.7)	-6.864	<.001	0.35	.023	.001	.654
	SDS-alcohol	8.5 (4.5)	5.4 (4.2)	-9.963	<.001	0.58	.042	.002	.474
	SDS-drugs	8.4 (3.3)	5.6 (4.1)	-7.258	<.001	0.55	.081	.007	.285
	PHQ	8.4 (5.8)	6.1 (5.5)	-6.380	<.001	0.59	.129	.017	.168
	GAD	9.6 (6.5)	5.7 (6.0)	-4.400	<.001	0.69	.162	.026	.311
**Females, n=169**									
	Quality of life	8.6 (4.3)	10.4 (4.3)	4.590	<.001	0.35	.098	.010	.206
	RPM	38.9 (13.4)	34.3 (14.5)	-3.741	<.001	0.29	.114	.013	.144
	SDS-alcohol	8.7 (4.5)	5.6 (3.9)	-6.968	<.001	0.61	.003	.001	.969
	SDS-drugs	8.8 (4.0)	6.4 (3.7)	-3.994	<.001	0.47	.127	.016	.288
	PHQ	8.4 (5.3)	5.6 (4.5)	-4.673	<.001	0.68	.098	.010	.206
	GAD	8.9 (6.4)	4.8 (3.3)	-3.419	.001	0.85	.114	.013	.144
**Males, n=223**									
	Quality of life	9.0 (4.67)	11.4 (4.5)	7.119	<.001	0.48	.057	.003	.401
	RPM	37.6 (14.8)	31.5 (16.5)	-5.851	<.001	0.40	.109	.012	.114
	SDS-alcohol	8.3 (4.6)	5.3 (4.4)	-7.148	<.001	0.55	.068	.005	.384
	SDS-drugs	8.2 (3.9)	5.0 (4.2)	-6.014	<.001	0.60	.041	.002	.679
	PHQ	8.5 (6.1)	6.5 (6.1)	-4.501	<.001	0.54	.089	.008	.468
	GAD	10.1 (6.5)	6.2 (7.3)	-2.900	.004	0.58	.180	.032	.389
**Alcohol, n=210**									
	Quality of life	8.9 (4.3)	10.4 (4.3)	4.851	<.001	0.34	.030	.001	.670
	RPM	38.1 (13.8)	34.6 (14.7)	-3.741	<.001	0.26	.025	.001	.728
	SDS-alcohol	8.4 (4.6)	5.8 (4.3)	-7.064	<.001	0.56	.067	.005	.397
	SDS-drugs	8.4 (4.0)	5.5 (1.1)	-5.155	<.001	0.53	.104	.011	.313
	PHQ	6.6 (3.5)	5.2 (3.7)	-3.153	.002	0.48	.220	.048	.157
	GAD								
**Non-opiate and crack, n=95**									
	Quality of life	8.3 (4.6)	11.4 (4.7)	5.778	<.001	0.59	.196	.039	.056
	RPM	39.6 (14.7)	31.8 (15.7)	-4.846	<.001	0.50	.041	.002	.693
	SDS-alcohol	9.1 (4.3)	5.5 (3.9)	-5.585	<.001	0.62	.081	.007	.474
	SDS-drugs	8.3 (3.6)	5.3 (3.8)	-3.223	.001	0.62	.206	.042	.302
	PHQ	10.9 (6.9)	7.2 (6.8)	-4.819	<.001	0.70	.146	.021	.326
	GAD	10.0 (6.3)	6.2 (6.3)	-3.669	<.001	0.71	.080	.006	.693
**Opiate and crack, n=88**									
	Quality of life	9.7 (4.8)	11.8 (4.5)	4.291	<.001	0.46	.011	.001	.922
	RPM	36.8 (14.7)	29.3 (17.4)	-3.462	.001	0.38	.005	.001	.965
	SDS-alcohol	7.7 (4.8)	4.4 (4.3)	-4.173	<.001	0.57	.135	.018	.322
	SDS-drugs	8.5 (4.0)	5.8 (4.2)	-4.053	<.001	0.56	.059	.004	.677
	PHQ	10.3 (7.2)	5.7 (5.2)	-2.861	.004	0.56	.034	.001	.869
	GAD	9.2 (6.6)	4.7 (5.4)	-2.450	.014	0.65	.280	.079	.332

^a^Changes in scores with time in weeks

### The Whole Sample

A significant increase in quality of life was found, along with significant decreases in both alcohol and drug dependence, depression, anxiety, and other areas of psychosocial impairment, as measured by the RPM (all *P*<.001). Proportions of all participant’s reaching cut-off scores decreased, including for; clinically significant drug dependence (92.9%-76.6%), alcohol dependence (86.6%-71.4%), depression (70.2%-50.0%), and anxiety (69.0%-46.3%). Effect sizes were large for drug dependence (*r*=0.55), depression (r=0.59), and anxiety (*r*=0.69), while medium effect sizes were found for quality of life (*r*=0.43) and RPM scores (*r*=0.35). Linear regressions revealed that time elapsed between baseline and follow-up assessment was not predictive of changes in scores.

### Females

A significant increase in quality of life was found for females, along with significant decreases in both alcohol and drug dependence, depression, and other areas of psychosocial impairment measured by the RPM (all *P*<.001). A significant decrease in anxiety (*P*=.001) was also observed. Proportions of female’s reaching cut-off scores decreased, including for; clinically significant alcohol dependence (85.4%-76.7%), drug dependence (93.3%-87.5%), depression (72.8%-53.2%), and anxiety (64.7%-50.0%). The effect size was very large for anxiety (*r*=0.85), large for alcohol dependence (*r*=0.61) and depression (*r*=0.68), medium for quality of life (*r*=0.35) and drug dependence (*r*=0.47), and small for RPM scores (*r*=0.29). Linear regressions revealed that time elapsed between baseline and follow-up assessment was not predictive of changes in scores.

### Males

A significant increase in quality of life was found for males, along with significant decreases in both alcohol and drug dependence, depression, and other areas of psychosocial impairment measured by the RPM (all *P*<.001). A significant decrease was also found in anxiety (*P*=.004). Proportions of male’s reaching cut-off scores decreased, including for; clinically significant alcohol dependence (87.6%-67.3%), drug dependence (92.5% to 68.9%), depression (68.2%-47.8%), and anxiety (71.8%-44.0%). Large effect sizes were found for alcohol (*r*=0.55), drug dependence (*r*=0.60), depression (*r*=0.54), and anxiety (*r*=0.58), and medium for quality of life (*r*=0.48) and RPM scores (*r*=0.40). Linear regressions revealed that time elapsed between baseline and follow-up assessment was not predictive of changes in scores.

### Alcohol as the Main Substance of Misuse

A significant increase in quality of life was found for service users citing alcohol as their main substance of misuse, along with significant decreases in both alcohol and drug dependence, and other areas of psychosocial impairment measured by the RPM (all *P*<.001). A significant decrease was observed in depression (*P*=.002), however, no data were available for this subgroup for anxiety. Proportions of alcohol misusers reaching cut-off scores decreased, including for; clinically significant alcohol dependence (85.8%-72.0%), drug dependence (92.9%-74.0%), and depression (65.0%-48.8%). Large effect sizes were found for alcohol (*r*=0.56) and drug dependence (*r*=0.53), medium for quality of life (*r*=0.34) and depression (*r*=0.44), and small for RPM scores (*r*=0.26). Linear regressions revealed that time elapsed between baseline and follow-up assessment was not predictive of changes in scores.

### Non-Opiate and Crack Users

A significant increase in quality of life was found for non-opiate and crack users, along with significant decreases in RPM scores, alcohol dependence, depression, and other areas of psychosocial impairment measured by the RPM (all *P*<.001). A significant decrease in drug dependence (*P*=.001) was also found. Proportions of non-opiate and crack users' reaching cut-off scores decreased, including for; clinically significant alcohol dependence (87.5%-77.5%), drug dependence (96.4%-88.9%), depression (78.9%-51.0%), and for anxiety (70.8%-48.1%). Large effect sizes were found for quality of life (*r*=0.59), RPM scores (*r*=0.50), alcohol (*r*=0.62), and drug dependence (*r*=0.62), and very large effect sizes for depression (*r*=0.70) and anxiety (*r*=0.71). Linear regressions revealed that time elapsed between baseline and follow-up assessment was not predictive of changes in scores.

### Opiate and Crack Users

A significant increase in quality of life was found for opiate (and opiate substitute medications) and crack users, along with significant decreases in alcohol and drug dependence (all *P*<.001), RPM scores (*P*=.001), depression (*P*=.004), and anxiety (*P*=.014). Proportions of opiate and crack users reaching cut-off scores decreased, including for; clinically significant alcohol dependence (87.7%-57.1%), drug dependence (91.1%-75.0%), depression (72.7%-50.0%), and anxiety (67.2%-42.9%). Large effect sizes were found for alcohol (*r*=0.57), drug dependence (*r*=0.56), depression (*r*=0.56), and anxiety (*r*=0.65), and medium effect sizes for quality of life (*r*=0.46) and RPM (*r*=0.38). Linear regressions revealed that time elapsed between baseline and follow-up assessment was not predictive of changes in scores.

## Discussion

### Principal Findings

This study sought to explore clinical outcomes in a heterogeneous group of service users accessing Breaking Free Online (BFO), an online CAT program for substance misuse. Due to the heterogeneous nature of the sample, in addition to looking at changes in the sample as a whole, subgroup analyses were also conducted to ascertain whether specific groups of service users benefited from using the program.

When the sample as a whole was included in the analyses, significant improvements in all aspects of psychosocial functioning were identified, and relatively robust effect sizes were found. Similar findings were obtained in all subgroups including females, males, service users reporting their primary problem substance as being alcohol, non-opiate and crack users, and opiate (and opiate substitute medications) users. Improvements were also seen in the severity of dependence on alcohol and drugs, reductions in depression and anxiety, improvements in quality of life, and reductions in the six domains of biopsychosocial functioning measured by the Recovery Progression Measure (RPM). The findings related to RPM outcomes are not surprising given the BFO program contains evidence-based techniques specifically included because they support improvements in functioning in the domains measured by the RPM. It is possible that the improvements in substance dependence severity, mental health, and quality of life reported could result from these improvements in biopsychosocial functioning, and also contribute to improvements in the domains of biopsychosocial functioning measured by the RPM. Further work is needed to more fully understand the interrelationships between the techniques contained within the BFO program and the various psychometric outcomes reported.

Additionally, linear regressions revealed that time elapsed between baseline and post-intervention follow-up assessments in weeks was not predictive of the degree of change in psychometric scores. This would indicate that the length of time engaged with the BFO program (in weeks) was not associated with the degree of improvement across the psychometric outcomes measured. This may reflect the fact that service users engaged with the program for the length of time they felt necessary, according to their self-perceived level of need in terms of intervention intensity requirement, or that only a brief period of engagement was sufficient for improvement, which is consistent with findings from studies with other brief interventions for alcohol [38,39]. Alternatively, this could also indicate that BFHJ is not subject to the ‘dosage’ effect that other interventions based on cognitive-behavioral therapy (CBT) principles are [40,41], which may be explained by the fact that BFO is modular rather than linear in nature. This means that users of the program can access the sections of the program that are most relevant to them, without having to work sequentially through content that may not be relevant. In other words, the program can be tailored to the needs of the individual. The literature suggests that the more capable a complex behavioral change intervention is of being tailored, the more likely it is to be effective [42].

“However, despite the encouraging findings when psychometrics scores were used to examine changes from baseline to post-intervention follow-up in a number of subgroups of service users, the findings were less impressive for the proportions of service users reaching cut-offs scores for clinically significant alcohol and drug dependence, depression and anxiety. Although for all subgroups the proportion of service users reaching cut-offs for clinically significant alcohol and drug dependence reduced between the baseline and post-intervention assessments, these reductions were not particularly large. The largest reduction was seen in the opiate and crack-using group who went from 87.7% (77/88) at baseline to 57.1% (50/88) at the post-intervention assessment, a reduction in 30.6% of service users in this group. The smallest decrease was seen in the female service user group with respect to the proportion reaching cut-off scores for clinically significant drug dependence (93.3%-87.5%), a reduction of just 5.8%.

The findings generated by this study have provided initial outcomes data to support the effectiveness of BFO with groups that have historically been less well represented in traditional substance misuse services, such as female service users. Women are a group of individuals who have been found to face a unique set of barriers to accessing support for substance misuse [14]. Many of these are associated with issues around finding and funding childcare, as many services have been described as being environments that are not particularly child-friendly [43]. Many women also cite concerns over social services involvement as a key barrier to approaching substance misuse services, and the additional stigma many women feel around the fact that their substance misuse may cause people to question their role as an effective caregiver, a role that has traditionally been seen as a female one [44,45]. Despite these additional barriers that women face when accessing support for substance misuse, and the underrepresentation of women in standard services, the sample in this study was quite evenly split, with 43.0% (169/393) of the sample being female. This is over twice the proportion seen in traditional substance misuse services, which is usually around 20%, with some variation according to the specific substance [46]. This may demonstrate that BFO, a completely confidential intervention that can be accessed privately at home, can provide a solution to some of the barriers women who misuse substances face when attempting to overcome their difficulties.

Another group who may wish to access support from the privacy of their own home is alcohol consumers who feel that accessing support via traditional services is associated with stigma [47]. As with women, this group of individuals may prefer to access support in private, rather than having to admit in a more open way that they have difficulties with alcohol, particularly as many people have concerns about the impact this may have on their professional and personal lives. This may also be the case for service users who, at first, used alcohol in a recreational manner, and yet may find that, in time, they became dependent. Although these groups may prefer interventions that can be used privately at home, it does not negate the potential of the kinds of interventions typically delivered in service environments to also be effective in the home environment [48,49].

With respect to the individuals that traditionally present at substance misuse services, which were those reporting they were dependent on opiates, opiate substitutes or crack, this subgroup had the same positive outcomes as in the other subgroups. However, the significance levels and effect sizes were not quite as strong as in the other groups, specifically in relation to quality of life and severity of depression and anxiety. This makes sense given this particular subgroup may have more deeply entrenched difficulties due to the substances on which they are dependent, and the additional complexity of addressing a physical and psychological dependence, as opposed to other substances that are primarily psychologically addictive. Additionally, opiate and crack-using individuals may be leading more chaotic lifestyles than other groups of substance dependent individuals, particularly given the criminal behaviors many need to engage in whilst attempting to financially support their habit [50].

### Limitations

The promising outcomes obtained in this study highlight an opportunity for traditional substance misuse services to market CAT as a targeted intervention that meets the needs of hidden and emerging drug using populations. Equally, the outcomes indicate that CAT may be a clinically effective intervention approach for a range of groups of substance dependent individuals that are not usually well represented in traditional substance misuse services. However, although the research reported here includes a relatively substantial sample size (N=393), the response rate was relatively low at 50.0% (393/785), and the work is still preliminary and exploratory, so there were some limitations that deserve consideration when drawing conclusions.

Firstly, the sample in the study were self-selecting and so could conceivably be assumed to be relatively motivated individuals, and hence more likely to reduce their alcohol and drug intake than service users who were not participating in the study. Therefore the potential impact of motivation in determining the outcomes obtained is not fully understood. It is also not known why non-participating service users did not provide follow-up assessment data; the reasons for this can only be speculative, although internet access could be one potential avenue for future research into why some services accessed BFO successfully and provide follow-up data, and others did not. Additionally, all data reported were based on service users’ self-reports of psychosocial functioning, and so may not have been entirely reliable.

It is also not known how relevant the findings from the study are to a wide range of ethnic groups, as the vast majority of participants in the study were White-British or White-Irish (95.9%, 377/393). However, this is representative of the demographic of service users accessing substance misuse services in the United Kingdom, in which approximately only 10% of service users are from black and minority ethnic (BME) communities [51]. BME communities are often described as hidden populations because of their underrepresentation within treatment services. There are multiple barriers to such BME communities accessing support via traditional substance misuse services such as lack of cultural sensitivity by the service, distrust of confidentiality, language barriers, stigma, and the failure of drug services to target minority ethnic drug users [52].

The BFO program could be used to engage members of BME communities as it can be delivered in any community setting away from traditional drug and alcohol services, such as places of residence, community halls, and religious buildings. As such, it can overcome the stigma of attending a traditional service. There is also a supporters section within BFO, which provides guidance to those who are supporting an individual using the programs to address their substance use. This supporter's guidance section could be used by individuals who may act as culturally appropriate supporters not associated with traditional drug and alcohol services, such as community elders, outreach workers, or other members of a community.

Another limitation lies in the fact that there was no control group included in the study. However, work is already underway that includes comparison controls, randomization to study groups, and a follow-up element to examine the long-term impact of BFO. Other work currently being conducted includes examining how different service users access the program in terms of time spent online and the specific outcomes that might be expected from accessing specific techniques within the program. Additionally, the possible additive effect of BFO when used in conjunction with ‘treatment as usual’ will also be examined as it is not known whether the outcomes obtained can be attributed to BFO, or whether the service users included in the present study may have improved due to the combined effect of BFO and other sources of support and treatment.

### Conclusions

This study demonstrated that BFO provides, at least in the short-term, a clinically effective intervention option for a wide range of service users accessing support for issues around dependence to a range of different substances. Additionally, the outcomes reported here are a first in terms of CAT approaches for substance misuse as they come from a sample of service users accessing support in real world substance misuse services, not a sample of participants in a highly controlled research study with limited ecological validity. Furthermore, as BFO provides confidential support that can be accessed outside of standard opiate and crack-centric services, it may enable some groups of individuals to overcome barriers that may prevent them from approaching services for support via more traditional interventions, with stigma being a significant obstacle for many. A comprehensive research program is currently underway to evaluate the program further, and it is hoped that as the evidence base for BFO increases, the program will be made even more widely available, enabling more individuals to access and use it in their recovery from substance dependence.

## Abbreviations

BFO: Breaking Free Online
CAT: computer-assisted therapy
CBT: cognitive behavioral therapy
GAD: General Anxiety Disorder Scale
LBM: Lifestyle Balance Model
NICE: National Institute for Health and Care Excellence
PHQ: Patient Health Questionnaire
RPM: Recovery Progression Measure
SDS: Severity of Dependence Scale
WHOQOL-BREF: World Health Organization Quality of Life assessment
